# Traffic-Related Air Pollution Exposure in the First Year of Life and Behavioral Scores at 7 Years of Age

**DOI:** 10.1289/ehp.1205555

**Published:** 2013-05-21

**Authors:** Nicholas C. Newman, Patrick Ryan, Grace LeMasters, Linda Levin, David Bernstein, Gurjit K. Khurana Hershey, James E. Lockey, Manuel Villareal, Tiina Reponen, Sergey Grinshpun, Heidi Sucharew, Kim N. Dietrich

**Affiliations:** 1 Division of General and Community Pediatrics, and; 2 Division of Biostatistics and Epidemiology, Cincinnati Children’s Hospital Medical Center, Cincinnati, Ohio, USA; 3 Department of Environmental Health, and; 4 Department of Medicine, Division of Immunology, Allergy and Rheumatology, University of Cincinnati College of Medicine, Cincinnati, Ohio, USA; 5 Division of Asthma Research, Cincinnati Children’s Hospital Medical Center, Cincinnati, Ohio, USA

**Keywords:** attention deficit/hyperactivity disorder, child behavior, epidemiology, land use regression, traffic-related air pollution

## Abstract

Background: There is increasing concern about the potential effects of traffic-related air pollution (TRAP) on the developing brain. The impact of TRAP exposure on childhood behavior is not fully understood because of limited epidemiologic studies.

Objective: We explored the association between early-life exposure to TRAP using a surrogate, elemental carbon attributed to traffic (ECAT), and attention deficit/hyperactivity disorder (ADHD) symptoms at 7 years of age.

Methods: From the Cincinnati Childhood Allergy and Air Pollution Study (CCAAPS) birth cohort we collected data on exposure to ECAT during infancy and behavioral scores at 7 years of age. Children enrolled in CCAAPS had at least one atopic parent and a birth residence either < 400 m or > 1,500 m from a major highway. Children were followed from infancy through 7 years of age. ECAT exposure during the first year of life was estimated based on measurements from 27 air sampling sites and land use regression modeling. Parents completed the Behavioral Assessment System for Children, 2nd Edition, when the child was 7 years of age. ADHD-related symptoms were assessed using the Hyperactivity, Attention Problems, Aggression, Conduct Problems, and Atypicality subscales.

Results: Exposure to the highest tertile of ECAT during the child’s first year of life was significantly associated with Hyperactivity *T*-scores in the “at risk” range at 7 years of age, after adjustment [adjusted odds ratio (aOR) = 1.7; 95% CI: 1.0, 2.7]. Stratification by maternal education revealed a stronger association in children whose mothers had higher education (aOR = 2.3; 95% CI: 1.3, 4.1).

Conclusions: ECAT exposure during infancy was associated with higher Hyperactivity scores in children; this association was limited to children whose mothers had more than a high school education.

Ultrafine particles (UFP; < 100 nm in diameter) are implicated in the pathophysiology of air pollution–related disease (Zanobetti and Schwartz 2009). Research in animals and humans demonstrates that the brain may be affected by traffic-related air pollution (TRAP), particularly UFP. Oberdörster and colleagues demonstrated translocation of inhaled ultrafine particles from the respiratory tract to extrapulmonary sites including the olfactory bulb in rats (Kreyling et al. 2002; Oberdörster et al. 2004). In an autopsy study, Calderón-Garcidueñas et al. (2008b) reported an association between exposure to high levels of ambient air pollution and histological changes in the brain consistent with neuroinflammation. Their study of otherwise normal children and young adults who died from accidents also demonstrated the presence of particulate matter in perivascular tissues of the frontal cortex. This group has also reported evidence of brain parenchymal changes on MRI (magnetic resonance imaging) and significant deficits in cognition, memory, and executive function in children exposed to high levels of ambient air pollution compared with low-exposure matched controls (Calderón-Garcidueñas et al. 2008a).

Epidemiologic studies have also reported associations between air pollution exposure and negative neurocognitive outcomes in school-age children (Suglia et al. 2008; Wang et al. 2009). Exposure to black carbon (a marker of TRAP and diesel exhaust) was associated with decreased cognitive scores for verbal and nonverbal intelligence and in memory at 8–11 years of age in a longitudinal cohort population of 202 children (Suglia et al. 2008). Exposure to traffic-related air pollution [measured by NO_2_ (nitrogen dioxide) and PM_10_ (particulate matter ≤ 10 µm in diameter)] was associated with poor performance in Visual Simple Reaction Time (both preferred and nonpreferred hands), Continuous Performance, Digit Symbol, Pursuit Aiming, and Sign Register assessments in a cross-sectional study of 861 children 8–9 years old (Wang et al. 2009). NO_2_ exposure, a surrogate for TRAP exposure was negatively associated with cognitive development at 4 years of age in 210 boys in a European birth cohort, although the association was not statistically significant (Freire et al. 2010). Prenatal exposure to polycyclic aromatic hydrocarbons was not associated with behavioral problems at 3 years of age (Perera et al. 2006), but was associated with attention problems and symptoms of anxiety/depression at 6–7 years of age in a U.S. cohort (Perera et al. 2012).

Attention deficit/hyperactivity disorder (ADHD) is a common psychiatric diagnosis in children characterized by symptoms of inattentiveness and/or hyperactivity that are present before 7 years of age in more than one setting, and are not explained by another cause (First 2000). ADHD affects 7–9.5% of children in the United States between 4 and 15 years old, or approximately 4.0 million children [Bloom and Cohen 2007; Braun et al. 2006; Centers for Disease Control and Prevention (CDC) 2010]. There is a strong familial tendency for ADHD, with heritability estimated to be as high as 75%; lower socioeconomic status, younger age, and male sex are associated with high prevalence of ADHD (Biederman and Faraone 2005). However, epidemiological studies suggest that both genetic and environmental factors are associated with the development of ADHD (Biederman and Faraone 2005; Braun et al. 2006). An analysis of 1999–2002 National Health and Nutrition Examination Survey (NHANES) data indicated that environmental tobacco smoke and lead were associated with ADHD (Braun et al. 2006). The impact of TRAP on childhood behavior and its potential association with ADHD is unknown, though a recent cross-sectional study reported an association between particulate matter air pollution (PM_10_) exposure and a DSM-IV (*Diagnostic and Statistical Manual of Mental Disorders, 4th Edition*; American Psychiatric Association 1994) diagnosis of ADHD among children 9–17 years old (*n* = 1819) in India (Siddique et al. 2011).

Associations between early-childhood exposures to environmental chemicals and subsequent neurodevelopmental disorders have been reviewed elsewhere (Grandjean and Landrigan 2006; Landrigan et al. 2005). Knowledge about the deleterious effects of lead, polychlorinated biphenyls, and mercury on children’s developing brains suggests that early-life exposures can have significant consequences for adverse neurobehavioral outcomes later in life (Grandjean and Landrigan 2006), but the impact of TRAP on neurobehavior is less well understood.

The objective of this study was to determine whether exposure to TRAP during early childhood is associated with behavioral outcomes at 7 years of age, particularly hyperactivity, attention problems, aggression, conduct problems, and atypicality—behaviors that are considered odd or strange (Reynolds and Kamphaus 2004) that often co-exist with ADHD (Cunningham and Jensen 2011; Volker et al. 2010).

## Materials and Methods

*Study design*. The sample for the present analysis was drawn from participants in the Cincinnati Childhood Allergy and Air Pollution Study (CCAAPS). Children enrolled in CCAAPS, a prospective birth cohort, were selected based on their residence at time of birth being either near (< 400 m) or far (> 1,500 m) from a major highway or bus route (LeMasters et al. 2006). From birth records, newborns were identified from the Cincinnati, Ohio, metropolitan area from 2001 through 2003. Parents who reported allergy symptoms and had a positive skin prick test to one of 15 aeroallergens were eligible to enroll their infant. Children completed annual clinical examinations at ages 1–4 and 7 years. At each of these visits a medical history, physical exam, and skin prick test were obtained. Parents also reported the locations where their child spent at least 8 hr/week during the previous year, including new residences, child care facilities, or relatives’ homes. The CCAAPS study was approved by the University of Cincinnati Institutional Review Board, and parents provided informed consent before their children were enrolled in the study.

*Child behavioral assessment*. At the 7-year study visit, parents completed a psychological assessment questionnaire, the Behavioral Assessment System for Children, Parent Rating Scale, 2nd Edition (BASC-2) (Reynolds and Kamphaus 2004). The BASC-2 consists of 160 questions that are answered by parents as never, sometimes, often, or always. It is designed to assess a child’s adaptive and problematic behaviors in both the community and home settings (Reynolds and Kamphaus 2004). The BASC-2 was validated for use on a U.S. school-age population. After the parent completed the BASC-2, it was scored using BASC-2 scoring software (BASC-2 PRQ ASSIST™). BASC-2 scores consist of composite and subscale *T*-scores with a mean (± SD) of 50 ± 10. All subscale scores were calculated, but Hyperactivity, Attention Problems, Aggression, Conduct Problems, and Atypicality subscales were selected for analysis *a priori* because these symptoms often co-exist with ADHD (Cunningham and Jensen 2011; Volker et al. 2010). The BASC-2 has three internal validity scores. The F (“Fake bad”) index assesses whether the parent rated the child in an overly negative fashion. A high score (> 6) on this scale suggests that the parent rated the child’s behavior more severely than expected and that the results should be interpreted with caution (Reynolds and Kamphaus 2004). The Response Pattern (R) index is a count of the number of times an individual item’s response differs from the previous item’s response. A high (> 125) or low (< 66) R index value suggests that parent was not attentive to content of the questions and that the results should be interpreted with caution. The Consistency index identifies situations where the parent provided different answers to questions that are usually answered similarly. A Consistency index score > 17 suggests that the results should be interpreted with caution. The scoring protocol for the BASC-2 allows for no more than two missing items per each rating scale.

BASC-2 *T*-scores were dichotomized into those in the “at risk” range or higher versus those below the “at risk” cut-off *T*-score of 59. The “at risk” designation is used to indicate those children who have the potential of developing behavioral problems on a given subscale and require careful monitoring (Reynolds and Kamphaus 2004).

*Estimation of TRAP exposure*. A complete description of the air sampling at monitoring stations and the land-use regression (LUR) methodology developed and used to estimate traffic exposure for CCAAPS children has previously been described (Ryan et al. 2007, 2008). Briefly, ambient air sampling was conducted on a rotating basis at 27 sampling sites in the greater Cincinnati area from 2001 through 2006, and the average daily concentration of elemental carbon was determined for each. The portion of sampled elemental carbon attributed to traffic (ECAT) was determined by positive matrix factorization and UNMIX receptor models (Hu et al. 2006; Sahu et al. 2011). Thus, ECAT serves as a marker of traffic exhaust, primarily diesel exhaust (Sahu et al. 2011). The final LUR model relating ECAT measured at the sampling sites to land-use and traffic variables had an *R*^2^ of 0.73 with elevation, truck traffic within 400 m, and length of bus routes within 100 m as significant independent variables (Ryan et al. 2007, 2008). A time-weighted average daily exposure to ECAT was estimated for each CCAAPS participant during the first year of life based on parental report of locations where the child spent at least 8 hr/week, on average. The average hours per day at each of these addresses was weighted by the proportion of time spent at that location during the first year of life (Ryan et al. 2008).

For this study, we used the average daily ECAT exposure over the first year of life as the primary exposure.

*Covariates*. Environmental tobacco smoke (ETS) exposure during pregnancy was estimated by parental report of the number of cigarettes smoked by the mother during each trimester of the pregnancy and averaged to estimate prenatal ETS exposure. Postnatal ETS exposure during the first year of life was also obtained via questionnaire and dichotomized into exposed and nonexposed based on parental report of any cigarettes smoked in the home (Biagini Myers et al. 2012). The age of the home was used as a surrogate for lead exposure and was dichotomized based on the year the home was built (< 1950 or ≥ 1950). Cotinine measurements were obtained for at least one hair sample collected during a study visit from year 1 through year 4. If more than one measure was available, the earliest obtained sample was used.

At the baseline visit, demographic information was collected. Maternal and paternal education were captured by five categories [did not finish high school, high school or GED (general equivalency diploma), some college or trade school (≤ 3 years), college (≥ 4 years), and graduate school] and dichotomized at completion of high school/GED or less, or some college/trade school and higher. Household income and insurance status (Medicaid) was obtained by caregiver report. Income was dichotomized at $30,000/year, and Medicaid status was dichotomized (yes/no). Race/ethnicity was defined as either African American or non–African American based on parental report. Mothers reported their duration of breastfeeding at the 1-year clinical examination, and it was dichotomized as either > 5 months or ≤ 5 months. Breastfeeding for > 20 weeks was negatively associated with ADHD symptoms and positively associated with executive functioning at age 4 years in a birth cohort of 500 children (Julvez et al. 2007a). Child care attendance (yes/no) during the first year of life was determined by parental report of their child spending time at a babysitter, child care, or a relative’s home.

*Statistical analysis*. We estimated the association between ECAT exposure and behavior problems using linear regression for continuous BASC-2 subscale scores and logistic regression for dichotomized BASC-2 subscale scores defining children “at risk.” Because of the skewed distribution of ECAT exposure and the relationship between the highest tertile of ECAT exposure and recurrent night cough previously reported in this cohort (Sucharew et al. 2010), ECAT exposure was dichotomized at the highest tertile versus the lower two tertiles. An exploratory analysis was also performed with ECAT and BASC-2 scores as continuous variables.

Potential model covariates were identified based on previous work within the CCAAPS cohort linking environmental exposures with outcomes, and on established risk factors for childhood behavioral disorders, including sex, race/ethnicity, maternal and paternal education, household income, Medicaid status, duration of breastfeeding, pre- and postnatal ETS exposure, hair cotinine, age of home, and child care attendance (Biederman and Faraone 2005; Braun et al. 2006; Julvez et al. 2007a, 2007b). Factors that predicted BASC-2 Hyperactivity subscale score in the “at risk” range with *p* < 0.1 based on chi-square tests and bivariate logistic regression were entered into multivariable models for each outcome. Colinearity among the potential covariates was also assessed using chi-square, Pearson correlation, analysis of variance, or Kruskal–Wallis, as appropriate. To avoid colinearity among several covariates that could serve as markers for socioeconomic status, only maternal education was chosen to represent socioeconomic status in the multivariable models. To evaluate the potential differential impact of this variable on hyperactivity scores, additional analyses were performed. An interaction term (maternal education × ECAT) was entered into the model and further explored by an analysis stratified by maternal education.

Statistical analysis was carried out using Epi Info 3.5.1 (CDC, Atlanta, GA) and JMP 9 (SAS Institute Inc., Cary, NC).

## Results

Of the 762 children initially enrolled in the study, 617 (81%) completed the clinical examination at 7 years of age, and of these, 599 of participants (97%) completed the BASC-2. Observations were excluded from the analyses if the F-index score was < 0, indicating a large number of missing items on the BASC-2 (*n* = 5); an F-index score > 6 (*n* = 3); a consistency score > 17 (*n* = 4); or an R-index score suggesting parental inattention to the questionnaire (*n* = 9). In addition, two children with missing data from the 1-year visit were excluded, leaving 576 children. Of these, 36 children (6%) changed residential locations between birth and the 1-year visit, and 377 children (65%) moved between the 1-year visit and the 7-year visit.

Compared with children excluded from the present analysis (*n* = 186), those included were more likely to have mothers with more than a high school education or GED and an annual household income ≥ $30,000, and were less likely to have prenatal cigarette exposure (Table 1). The percentages of African-American children and children with homes built before 1950 were lower but not significantly different from the excluded children. The average estimated ECAT exposure was the same in both groups.

**Table 1:** Demographic characteristics and environmental exposures for children either included or excluded from study analysis.

Characteristic	Included (*n*=576)		Excluded (*n*=186)	
	*n* (%)	Mean±SD	*n* (%)	Mean±SD
Demographic characteristics
Age when BASC-2 administered (years)	576	6.9±0.3		NA
African American	117 (20.3)		49 (26.3)	
Male	317 (55.0)		98 (52.7)	
Mother’s education level (high school/GED or less)*	119 (21.3)		66 (35.9)	
Family income <$30,000*	131 (23.5)		71 (38.6)	
Environmental exposures
ECAT exposure, year 1	576	0.4±0.1	186	0.4±0.1
Hair cotinine, years 1–4*	451	0.2±0.3	123	0.2±0.4
Prenatal cigarette exposure*	57 (10.5)		33 (18.2)	
Cigarette exposure, year 1	110 (21.6)		47 (29.2)	
Home built before 1950	134 (26.9)		38 (36.2)	
Past medical history
Any positive SPT in ≥ 2 years	260 (45.1)			
Any ADHD medications, year 7	17 (3.0)			
BASC-2*T*-scores, year 7 (continuous)
Hyperactivity	576	50.8±10.3		
Attention Problems	576	50.9±10.2		
Aggression	575	50.3±9.6		
Conduct Problems	576	50.8±10.6		
Atypicality	576	49.4±9.5		
BASC-2*T*-scores “at-risk” range^*a*^
Hyperactivity	106 (18.4)			
Attention Problems	111 (19.3)			
Aggression	90 (15.7)			
Conduct Problems	81 (14.1)			
Atypicality	82 (14.3)			
Abbreviations: NA, not applicable; SPT, skin prick test. ^***a***^At-risk range=*T*-score >59. *p<0.05 comparing children included and excluded from analysis; values in the row are significantly different from each other.				

Of the 576 children, 55% were male and 20.3% were African American. Maternal education was at the level of high school/GED or less for 21.3%. Eleven percent reported tobacco use during pregnancy, whereas 21.6% reported tobacco exposure during the first year of life. The mean ± SD age when the BASC-2 was administered was 6.9 ± 0.3 years. Mean *T*-scores (± SD) for selected subscales were, for Hyperactivity, 50.8 ± 10.3; Aggression, 50.3 ± 9.6; Conduct Problems, 50.8 ± 10.6; Atypicality, 49.4 ± 9.5; and Attention Problems, 50.9 ± 10.2. Our sample *T*-score means ± SD were comparable to the expected *T*-score means (50 ± 10) based on the BASC-2 validation studies (Reynolds and Kamphaus 2004). The “at risk” range for each BASC-2 subscale is defined as scores ≥ 1 SD from the mean. The percentage of children in the “at risk” range for this study is as follows: Hyperactivity, 18.4%; Attention Problems, 19.3%; Aggression, 15.7%; Conduct Problems, 14.1%; and Atypicality, 14.3%. The percentage of children with either a Hyperactivity *T*-score or Attention Problems *T*-score in the clinical range for ADHD (> 69) was 9.2%.

The mean ECAT exposure estimate was 0.4 ± 0.1 µg/m^3^ (median = 0.35 µg/m^3^, 33rd percentile = 0.32 µg/m^3^, 66th percentile = 0.40 µg/m^3^). African-American race/ethnicity, having a home built < 1950, maternal education ≤ high school/GED, family income < $30,000, breastfeeding < 5 months, and hair cotinine were positively associated with ECAT (all *p* < 0.001). Age of the child’s home and hair cotinine were not considered for the final model because they were not significant predictors of the Hyperactivity BASC-2 subscale score in the “at risk” range. Of the 451 available hair cotinine samples, 18.4% were below the limit of detection (0.02 ng/mg). For those samples below the limit of detection, imputed values were entered into the model (Hornung and Reed 1990). In addition, we did not adjust for race/ethnicity because it was not a significant predictor of risk for a hyperactivity *T*-score in the “at risk” range. For parsimony, only maternal education was chosen to represent socioeconomic status because it was significantly associated with income and Medicaid status. Maternal education was also significantly associated with duration of breastfeeding as well as an at-risk range *T*-score for hyperactivity.

Unadjusted linear regression models of BASC-2 *T*-scores in association with ECAT as a continuous variable indicated positive association with all subscale *T*-scores except Aggression, though the association was statistically significant for Atypicality only (Table 2). Scores indicating that children were at risk were significantly associated with high versus low ECAT exposure (≥ 0.40 vs. < 0.40 µg/m^3^) for Hyperactivity, Conduct Problems, and Atypicality based on unadjusted logistic models (all *p* < 0.01) (Table 2). After adjusting for sex, cigarette exposure during the first year of life, and maternal education, high ECAT remained significantly associated with an “at risk” score for Hyperactivity only [adjusted odds ratio (aOR) = 1.7; 95% CI: 1.0, 2.7]. An ECAT × maternal education interaction term was added to this model but was not statistically significant (*p* = 0.07).

**Table 2:** Association between BASC-2 *T*‑scores and ECAT exposure during the first year of life among 576 children.

BASC-2 subscale	ECAT (µg/m^3^) and continuous score^*a*^ [β (95% CI)]	Total in “at risk” range (*n*)	“At risk” with high ECAT exposure (*n*)	ECAT (dichotomous)^*b*^^**^and “at risk” score, unadjusted [OR (95% CI)]	ECAT (dichotomous)^*b*^ and “at risk” score, adjusted^*c*^ [OR (95% CI)]
Hyperactivity	5.1 (–1.2, 11.4)	106	48	1.9 (1.2, 2.9)**	1.7 (1.0, 2.7)*
Attention Problems	4.3 (–1.8, 10.4)	111	44	1.4 (0.9, 2.2)	1.1 (0.6, 1.7)
Aggression^*d*^	0.0 (–5.7, 5.7)	90	37	1.5 (0.9, 2.4)	1.2 (0.7, 2.0)
Conduct Problems	6.1 (–0.2, 12.4)	81	39	2.1 (1.3, 3.3)**	1.5 (0.9, 2.6)
Atypicality	6.8 (1.1, 12.5)*	82	39	2.0 (1.3, 3.2)**	1.5 (0.9, 2.6)
^***a***^Association between BASC-2 subscale score (continuous) and a 1-µg/m^3^ increase in ECAT, unadjusted linear regression model.^***b***^Association between “at risk” BACS-2 subscale score and high vs. low ECAT (≥40 µg/m^3^ vs. <0.40 µg/m^3^), logistic regression model.^***c***^Adjusted for sex, report of ETS exposure in the first year of life, and maternal education. In the adjusted analysis, those missing maternal education (*n*=18) and ETS exposure (*n*=66) were excluded (total*n*=504).^***d***^Due to missing data for one child with lower ECAT exposure,*n*=575 for the continuous analysis and unadjusted OR,*n*=503 for the adjusted OR. *p<0.05. **p<0.01.					

Stratification by maternal education modified the relationship between ECAT exposure and hyperactivity. Higher ECAT exposure was associated with a significant increase in Hyperactivity score only among those children whose mothers had more than a high school education (Table 3).

**Table 3:** ORs (95% CIs) from logistic regression for associations between “at risk” scores for ADHD-related BASC-2 subscales^*a*^ and high versus low ECAT exposure during the first year of life,^*b*^ stratified by maternal education high school or less vs. more than high school.

BASC-2 scale	Lower maternal education*n*=119				Higher maternal education*n*=439			
	*n*^*c*^	Unadjusted [OR (95% CI)]	Adjusted^*d*^ [OR (95% CI)]	*p*-Value	*n*^*c*^	Unadjusted [OR (95% CI)]	Adjusted^*d*^ [OR (95% CI)]	*p*-Value
Hyperactivity	18	0.9 (0.4, 1.9)	0.9 (0.4, 2.2)	0.83	29	2.4 (1.4, 4.1)*	2.3 (1.3, 4.1)*	0.003
Attention Problems	20	0.8 (0.4, 1.7)	0.8 (0.3, 1.8)	0.97	21	1.4 (0.8, 2.4)	1.3 (0.7, 2.4)	0.40
Aggression	13	0.7 (0.3, 1.7)	0.6 (0.2, 1.7)	0.34	24	1.9 (1.1, 3.4)*	1.5 (0.8, 2.8)	0.16
Conduct Problems	17	1.3 (0.6, 2.9)	1.1 (0.4, 2.8)	0.88	21	2.2 (1.2, 3.9)*	1.7 (0.9, 3.2)	0.14
Atypicality	18	1.4 (0.6, 3.2)	1.6 (0.6, 4.1)	0.31	17	1.7 (0.9, 3.2)	1.4 (0.7, 2.8)	0.30
^***a***^BASC-2*T*-score >59.^***b***^ECAT ≥40 µg/m^3^ vs. <0.40 µg/m^3^.^***c***^Number with “at risk”*T*-score and high ECAT.^***d***^Adjusted for sex, and age 1year cigarette exposure. *p <0.05.								

## Discussion

To our knowledge, this is the largest prospective cohort with the longest follow-up investigating early life exposure to TRAP and neurobehavioral outcomes at school age. We observed an association between ECAT exposure and BASC-2 Hyperactivity scores that appeared to be limited to children whose mothers had higher education.

Several biological mechanisms could explain the association between hyperactive behaviors and exposure to TRAP. In an autopsy study comparing children and adults who lived in a cities with either high (*n* = 35) or low (*n* = 12) ambient air pollution [based on PM_2.5_ (PM with diameter ≤ 2.5 µm)], those in the highly exposed city (Mexico City) experienced significantly increased levels of inflammatory mediators and vasoconstrictors (Calderón-Garcidueñas et al. 2008b). Neuroinflammatory changes were observed in the highly exposed group, as evidenced by upregulation of cyclooxygenase-2 and interleukin-1β in the frontal cortex (Calderón-Garcidueñas et al. 2008b). Using electron microscopy, these investigators also noted the presence of UFP within erythrocytes within the capillaries of the frontal cortex in one subject (Calderón-Garcidueñas et al. 2008b). In a rat model, there is evidence of translocation of ^13^C nanoparticles (in the UFP size range of 36 nm diameter) through the olfactory nerves (Oberdörster et al. 2004) and this is one hypothesized route into the brain. Dysfunction of frontal-cortical circuits is associated with ADHD (Biederman and Faraone 2005). Diesel exhaust particles were selectively toxic to dopaminergic neurons in a rat tissue culture system (Block et al. 2004). Although the exact biological mechanism for ADHD has not been identified, there is evidence implicating the dopaminergic system in its pathophysiology (Biederman and Faraone 2005; Froehlich et al. 2007; Kahn et al. 2003; Swanson et al. 2007).

The association between high versus low ECAT and hyperactivity scores was limited to children whose mothers had more than a high school education. An analysis of the Multimodal Treatment Study of Children with ADHD (MTA) demonstrated a positive association between early diagnosis of ADHD and later poor school achievement (Langberg et al. 2011). One possible explanation for our finding is that lower school achievement in mothers is associated with maternal ADHD, and this predisposition to ADHD symptoms is a stronger predictor of hyperactivity in their children than is ECAT exposure. In addition, mothers with higher education may expect higher achievement, and this expectation may increase the parental report of behavioral concerns.

Other studies have demonstrated associations between markers of air pollution and neurobehavior in children. A positive association between residential proximity to freeways and autism has been reported in a study of 304 autism cases and 259 typically developing controls (Volk et al. 2011). In our study, we found an association between a marker of high TRAP exposure and atypical behaviors—the tendency to behave in ways that are immature or age-inappropriate, or considered odd and often associated with hyperactivity (Reynolds and Kamphaus 2004). This association remained positive but was attenuated and nonsignificant after adjusting for covariates. In a cohort of children from Menorca, Spain, nitrogen oxides (NO_x_) exposure from indoor cooking was associated with inattention behaviors but not hyperactivity (Morales et al. 2009). We found the opposite: no association between ECAT exposure and inattention, but a positive association between ECAT and hyperactivity. There are important differences in the constituents of indoor and outdoor air pollution, with indoor NO_x_ contribution coming mostly from indoor cooking and ECAT coming from traffic (Levy et al. 2010). A limitation of our study is that we do not have detailed data on indoor air pollutants, nor did we model NO_x_ in the outdoor air.

There are several other study limitations. One is that the population was selected on the basis of being at high risk for atopy, and therefore our findings may not be generalizable. However, the percentage of children with BASC-2 scores that would meet clinical criteria for ADHD was close to national estimates for ADHD prevalence. The percentage of children with either hyperactivity or inattention scores in the clinical range (9.2%) suggests that our study population is similar to the prevalence of ADHD in the general population (9.5%) (CDC 2010). In addition, associations with components of TRAP other than ECAT, including polyaromatic hydrocarbons, volatile organic compounds, and gaseous pollutants, were not assessed.

In our study, childhood exposure to TRAP was estimated by applying an LUR model to locations where caregivers reported living and spending time to estimate average daily exposure to ECAT. This LUR model was developed using average daily concentrations of ECAT sampled at 27 sites in the greater Cincinnati airshed from 2001 through 2006 (Hu et al. 2006; Ryan et al. 2007; Sahu et al. 2011). The strength of the LUR approach is its ability to characterize exposure to pollutants with high spatial variability. A limitation to our approach, however, is that temporal variability in exposure estimates depends on changes in residential or other locations. Thus, we are not able to distinguish the impact of prenatal from early childhood exposures because 94% of families remained in the same residential location from the child’s birth to 1 year of age.

Another potential limitation of the study is the lack of data regarding the behavioral health history of the CCAAPS families, including maternal hyperactivity. Childhood lead exposure is associated with ADHD (Braun et al. 2006; Froehlich et al. 2009), but blood lead levels were not measured among the children in this study. As a surrogate, we examined the age of the home, because most lead poisoning is children is caused by exposure to deteriorating paint in older buildings. However, we did not adjust for age of the home because it was not a significant predictor of Hyperactivity *T*-scores. Also, it is likely that ECAT is associated with higher traffic noise, so confounding by noise may bias associations with ECAT.

Among the strengths of this study is that it is a longitudinal birth cohort and that the exposure to ECAT is well characterized. The BASC-2 is a carefully validated instrument that provides scores that are applicable to a clinical setting. Finally, the population of the greater Cincinnati metropolitan area tends to be stable, and 79% of the original population completed the BASC-2. Children who completed the study did not differ in their estimated exposure to ECAT during the first year of life from those who did not complete the study, but their mothers were significantly more likely to have higher education levels.

## Conclusions

Although the prevalence of ADHD in children is increasing (CDC 2010), little is known about potential environmental contributions to its development. Compared with children exposed to ECAT < 0.40 µg/m^3^ during their first year of life, children with high ECAT exposure were 70% more likely to have a high Hyperactivity score after adjustment for potential confounders, with a slightly stronger association among children whose mothers had more than a high school education. Examination of age at exposure as well as gene–environment interactions might also inform understanding of the pathophysiology of traffic exposure and behavior. Given that 35.2 million people in the United States live in counties in nonattainment of their PM_2.5_ levels (U.S. Environmental Protection Agency 2009), approximately 11% of the U.S. population lives within 100 m of a four-lane highway (Brugge et al. 2007), and 40% of children attend school within 400 m of a major highway (Appatova et al. 2008), the observed association between TRAP and hyperactivity may have far-reaching implications for public health.
